# Reactivation of Ocular Toxoplasmosis in Non-Hispanic Persons, Misiones Province, Argentina

**DOI:** 10.3201/eid2205.150025

**Published:** 2016-05

**Authors:** Marcelo Rudzinski, Marina Khoury, Cristobal Couto, Daniel Ajzenberg

**Affiliations:** Universidad Católica de las Misiones, Misiones, Argentina (M. Rudzinski);; Instituto de Investigaciones Médicas Alfredo Lanari, Buenos Aires, Argentina (M. Khoury);; Hospital de Clínicas José de San Martin, Buenos Aires (C. Couto);; Université de Limoges, Limoges, France (D. Ajzenberg)

**Keywords:** *Toxoplasma gondii*, ocular toxoplasmosis, reactivation, retinochoroiditis, Hispanic, non-Hispanic, Native Americans, Argentina, South America, parasites

**To the Editor:** Ocular toxoplasmosis (OT), caused by the parasite *Toxoplasma gondii*, is known to be a major health problem in South America, especially in Colombia and Brazil (*1*–*3*). The highest prevalence of OT has been reported in Erechim, Rio Grande do Sul, Brazil, a state that borders Uruguay and Argentina, where a representative population-based household survey showed that 17.7% of 1,042 adults examined had OT (*1*). For comparison, it is estimated that ≈2% of *T. gondii*–infected persons in the United States manifest OT (*4*). It is still unclear whether the high rate of OT in South America is attributable to host or parasite genetic factors or differences in exposure rate (*5*,*6*).

In the province of Misiones in Argentina, the prevalence of OT in patients seeking care in an ophthalmic office is also high and has been documented as high as 20% (*7*). The area was mostly settled in the early 20th century by non-Hispanic European immigrants from Germany and Slavic countries who arrived through Southern Brazil.

We explored the relationship between ethnic origin and frequency of reactivation toxoplasmic retinochoroiditis (RTR) in patients who sought care in a private secondary care eye clinic in Oberá, Misiones, Argentina, during February 2004–May 2014. All patients with uveitis were examined by a single uveitis specialist (M.R.), who performed complete ophthalmological examinations, including visual acuity, anterior biomicroscopy, tonometry, and indirect ophthalmoscopy. Study inclusion criteria were presence of RTR in 1 or both eyes and specific *T. gondii* IgG in blood serum samples. A minimum of 3 months with no signs of intraocular inflammation was required to differentiate chronic active retinochoroiditis from 2 consecutive episodes of RTR.

The study included 112 nonimmunosuppressed patients with RTR. The patients completed a questionnaire including demographic data for the patient, as well as the first and last names and countries of origin of parents, grandparents, and great-grandparents. Informed consent was obtained from all participants and the study was approved by the human subjects review committee of Misiones Province. 

The patients were divided into 4 groups (Table): 1) patients reporting >1 ancestor born in Spain were considered Hispanic (n = 29); 2) patients without Hispanic ancestry who had >1 ancestor born in Poland, Ukraine, Russia, or Belarus, and who spoke Polish, Ukrainian, or Russian were considered Slavic (n = 28); 3) patients without Hispanic or Slavic ancestry who had >1 ancestor born in Germany or the Austro-Hungarian Empire and who spoke German were considered Germanic (n = 46); and 4) patients who did not fulfill the criteria of any of the above*-*mentioned groups were designated as others (n = 9). All patients had RTR at baseline; 28 had >1 more RTR episode during the follow-up period (Table). Multiple logistic regression analysis, in which Hispanic patients were used as a reference group, showed that Germanic and Slavic patients had a higher risk for reactivation during the follow-up period, but the odds ratio was significant only for Slavic patients after adjustment for rural dwelling.

**Table:** Demographic baseline characteristics of patients with reactivation of OT and multiple logistic regression analysis of the association between ethnic origin and risk for RTR during follow-up, Misiones province, Argentina, 2004–2014*

Demographic and follow-up data	Ethnicity			
	Hispanic, n = 29	Slavic, n = 28	Germanic, n = 46	Other, n = 9
Demographic data				
Male sex†	13 (44.83)	14 (50)	20 (43.48)	5 (55.56)
Median age, y (range)†	31 (6–67)	32.5 (14–70)	31 (6–71)	29 (6–68)
Rural dwelling†	18 (62.07)	23 (82.14)	30 (65.22)	5 (55.56)
Follow-up				
Length, median mo†	37	42	38.5	27
≥1 reactivation toxoplasmic retinochoroiditis	4 (13.79)	10 (35.71)	12 (26.09)	2 (22.22)
OR (95% CI)	Reference	3.47 (0.93–12.85)‡	2,2 (0.63–7.65)†	1.78 (0.27–11.86)†
OR adjusted for rural dwelling (95% CI)	Reference	4.07 (1.05–15.68)§	2.2 (0.65–8.01)†	1.73 (0.26–11.64)†

*Data are no. (%) patients, unless otherwise indicated. Analysis was performed using IBM SPSS Statistics (IBM, Armonk, NY, USA). p values <0.05 were considered significant. OR, odds ratio; OT, ocular toxoplasmosis; RTR, reactivation toxoplasmic retinochoroiditis. †p>0.05. ‡p = 0.06. §p = 0.04.

More than 95% of *Toxoplasma gondii* strains in Europe belong to the clonal type II lineage, whereas strains from South America are genetically divergent and diverse (*6*,*8*). We hypothesize that the European population is poorly adapted to South American strains and therefore more susceptible to OT. If this hypothesis is true, Native Americans who had a long history of exposure to atypical strains from South America should be more resistant to OT. This hypothesis is reinforced by a recent survey conducted among Mbyá-Guarani Indians, who had a serologic prevalence of toxoplasmosis 70%, but only 3.5% of them had toxoplasmic retinochoroidal lesions (M. Rudzinski, unpub. data).

Argentineans have a large incidence of European genetic heritage in their Y-chromosomal and autosomal DNA, but ≈50% of their mitochondrial gene pool is of Native American ancestry (*9*). The amount of admixture between Europeans and Native Americans with inheritance of resistance genes to OT from Native Americans may explain the difference of susceptibility to RTR between Hispanic and non-Hispanic Europeans. Admixture events between Europeans and Native Americans mainly involved Hispanics whose migration to Argentina started in the 16th century and continued until the mid*-*20th century. Persons in Argentina who have Spanish surnames can carry as much as 80% Native American genetic ancestry (*9*,*10*). In contrast, the Slavic and German Europeans migrated to Misiones only during a large surge of European immigration between 1890 and 1950, and did not have substantial admixture with Native Americans. Despite the fact that this study was not a random or representative sample of all ethnic groups in Argentina and the Native American mixture of the patients was not known, and environmental and dietary influences were not examined, our results suggest host genetic factors as determinants of disease severity in OT.
